# Comparisons of Science Motivational Beliefs of Adolescents in Taiwan, Australia, and the United States: Assessing the Measurement Invariance Across Countries and Genders

**DOI:** 10.3389/fpsyg.2021.674902

**Published:** 2021-08-02

**Authors:** Pey-Yan Liou, John J. H. Lin

**Affiliations:** ^1^Department of Education, Korea University, Seoul, South Korea; ^2^Graduate Institute of Science Education, National Taiwan Normal University, Taipei, Taiwan

**Keywords:** science motivational beliefs, TIMSS, gender differences, measurement invariance, cross-national comparisons

## Abstract

This study utilized international, large-scale assessment data to compare science motivational beliefs of adolescents within and between countries and genders. The study focused on the beliefs about science of eighth graders, including their self-concept in science, the intrinsic value they ascribed to science, and their beliefs about the utility of the subject. The study data were derived from the Trends in International Mathematics and Science Study in 2019 (TIMSS) that was conducted in Taiwan, Australia, and the United States. To ensure the validity of mean cross-group comparisons, the measurement invariance (MI) of the constructs was first assessed. The multiple-group confirmatory factor analysis and latent factor mean comparisons were applied to the data. The results indicated that the MI of science motivational beliefs across the three countries attained only metric invariance, rendering a latent mean comparison implausible. However, the cross-gender MI within each country attained scalar invariance, supporting the comparison of means across genders. The science motivational beliefs of females were significantly lower than those of males, with the exception of beliefs of US students about their utility value. The findings of this study raise concerns about the validity of current international comparisons of science motivational beliefs of the students while supporting the use of TIMSS data to identify gender differences in science motivation within each country. The implications of MI across countries and genders are discussed, and the importance of establishing MI is highlighted. The findings affirm that gender disparities in science motivational beliefs can be compared using constructs with sound psychometric properties.

## Introduction

Science motivational beliefs of students are crucial psychological measurements of learning and preferences for majors and careers in the fields of science, technology, engineering, and mathematics (STEM). While gender^1^ differences in science achievement during schooling have been narrowed to a negligible magnitude, the proportion of females in STEM-related professions remains lower than that of males (Mullis et al., 2020; World Economic Forum, 2020). Compared with the achievement in the subject, the motivational beliefs of students about science are viewed as more critical determinants for such gender disparities in the STEM fields (Wang and Degol, 2017), recognizing the wider point that adolescence is a crucial period of forming beliefs when planning for the future (Erikson, 1968). Understanding the extent to which there exist gender differences in science motivational beliefs should thus be the first step toward developing appropriate educational policies and interventions.

To draw valid conclusions regarding gender differences in science motivational beliefs of adolescents, the measurement invariance (MI) of the relevant constructs across groups must be established. Several studies (e.g., Liou and Liu, 2015; Ghasemi and Burley, 2019; Liou et al., 2020; Mejía-Rodríguez et al., 2020) claim to have identified the differences in motivational beliefs of students across groups (e.g., countries and genders); however, none has successfully demonstrated the equivalence of the scales before comparing the large-scale differences. In fact, the authors of these studies (Liou and Liu, 2015; Ghasemi and Burley, 2019) and other scholars (e.g., He et al., 2019) have raised concerns about the failure of researchers to check MI before making comparative inferences. Thus, the present investigation intended to focus on this gap by first determining the cross-group comparability of the data on science motivational beliefs before proceeding to substantive issues regarding gender differences.

The use of existing and reliable international large-scale assessment (ILSA) data is an effective and practical means of tracking the representative pattern of science motivational beliefs of adolescents. One of the major ILSA datasets, the *Trends in International Mathematics and Science Study* (TIMSS), has periodically investigated science motivational beliefs of eighth graders in numerous countries. This study used TIMSS data to examine gender disparities in science motivational beliefs and to assess the assumption of MI before examining such gender differences in Taiwan, Australia, and the United States.

### Theoretical Background of Science Motivational Beliefs of Adolescents and the Cultural Contexts

To conceptualize the science motivational beliefs of the students, the framework of the expectancy-value theory (EVT) was utilized. EVT is a comprehensive theoretical framework for explaining the relations between academic motivational beliefs and achievement-related outcomes (Eccles et al., 1983). EVT is mainly composed of two major elements, namely, expectancies for success (e.g., self-concept) and task values (e.g., intrinsic value and utility value). Self-concept refers to the subjective judgment of individuals of their selves formed through experience and evaluative feedback compared with their peers (Marsh and Shavelson, 1985; Bong and Clark, 1999). The task values are composed of four components, namely, intrinsic value, utility value, attainment value, and cost. Within the TIMSS 8th Assessment Framework (Mullis and Martin, 2017), only two of the four components (i.e., intrinsic value and utility value) were measured, so only these two could be examined in the present study. The intrinsic value is defined as engagement in activities for their own sake, i.e., for the inner enjoyment and satisfaction derived from taking part. In contrast, utility value refers to the usefulness of a task for future goals of the individuals, which are considered as the ends of the task (Wigfield and Eccles, 1992; Eccles and Wigfield, 2002). The three-factor structure composed of self-concept, intrinsic value, and utility value for motivational beliefs of adolescents, thus framing this investigation. Motivational beliefs are domain-specific (Eccles and Wigfield, 2002), so the present study focuses only on the science motivational beliefs.

The formation of the motivational beliefs of adolescents around science is deeply rooted in cultural contexts (Wigfield and Eccles, 1992; Liou, 2017), and the pattern of the motivational beliefs of adolescents is likely to vary across countries. The availability of TIMSS data from multiple countries potentially enables science motivational beliefs of adolescents across countries to be compared. To test the comparability, the present investigation focused on the data from three high-achieving countries, namely, Taiwan, Australia, and the United States. Among the 39 countries that participated in the most recent TIMSS assessment of eighth graders in 2019, Taiwan ranked second, Australia ranked ninth, and the United States ranked 11th. Their average scores were all significantly higher than the international average (Mullis et al., 2020). After taking the cognitive science achievement of adolescents into account, the results regarding equivalence or inequivalence of their motivational beliefs across countries could be pronounced. Additionally, the three countries have been top-ranked in terms of publications in leading science educational journals (e.g., the *International Journal of Science Education* and the *Journal of Research in Science Teaching*) for the past two decades (Lin et al., 2019). The findings of this study are therefore of value to stakeholders internationally and to researchers with a particular interest in Taiwan, Australia, and the United States.

### Gender Differences in Science Motivational Beliefs of Adolescents

The existence of gender disparities in the field of STEM is well-established. Riegle-Crumb and King (2010) analyzed US national data and found that, regardless of race, males dominated STEM majors in college and related occupations. The *Global Gender Gap Report* 2020 (World Economic Forum, 2020) showed the low percentage of female workers in STEM fields such as data analysis and AI, engineering, and cloud computing, all of which are at the frontiers of the new economy. However, the gender gap in science and mathematics achievement during schooling has narrowed to a negligible magnitude (O'Dea et al., 2018; Mullis et al., 2020), which suggests that it is unfeasible to attribute gender disparities in the STEM fields to cognitive factors, such as science achievement. In contrast, non-cognitive factors, such as the science motivational beliefs of adolescents, may constitute a key determinant of this gap. In fact, gender is one of the crucial characteristics associated with the science motivational beliefs of adolescents. Recent research has indicated that males tend to hold more positive science motivational beliefs than do females in most countries (Marsh et al., 2013; Wang and Degol, 2013). Thus, understanding the extent of gender differences in science motivational beliefs during adolescence is a critical step in designing appropriate educational interventions to close the gap that appears to exist.

While gender differences in science motivational beliefs of the students have long been a substantive topic of research, the majority of extant studies have not demonstrated scalar equivalences for males and females prior to conducting gender-based comparisons of quantitative data. Given that the membership of sociocultural groups could affect the conceptualization of underlying constructs, or the scores of scales (Byrne and van de Vijver, 2010), testing whether the means can be compared across groups appears to be a prerequisite step. Furthermore, Ghasemi and Burley (2019) and He et al. (2019) suggested that MI should be checked before proceeding to deeper analyses of TIMSS data. Establishing invariance in motivational beliefs would be helpful to exclude the possibility that gender differences do not relate to the differences in response tendencies between groups (Vandenberg and Lance, 2000). Therefore, more studies should focus on filling this gap in our understanding and strengthening the use of well-evaluated items to explore science motivational beliefs across groups.

### Measurement Invariance

MI concerns the extent to which the measurement is consistent across groups. The focus of MI is to ensure that a given scale measures the same trait in all groups (Schmitt and Kuljanin, 2008). Meredith (1993) defined the types of MI, in ascending order of rigor, as configural invariance, metric invariance, scalar invariance, and uniqueness invariance. To compare any scale-based scores, the scale must be assumed to measure the same trait in all groups. If that assumption is valid, comparisons and analyses of those scores, such as comparing factor scores among groups, are acceptable, permitting meaningful interpretations based on the results to be made (Vandenberg and Lance, 2000). Thus, the MI should reach at least the level of scalar invariance before comparing latent means across groups.

MI can be examined using the item-response theory approach (Halamová et al., 2019) or can be tested within a structural equation modeling (SEM) framework (Yuan and Chan, 2016; Toro et al., 2020). In the present study, the multiple-group confirmatory factor analysis (MGCFA) in the SEM framework was used, as SEM is pervasively used in the ILSA data analysis (e.g., Marsh et al., 2013).

The MGCFA model with structured means can be used to investigate the MI by testing a sequence of models, beginning with an unconstrained model and introducing equality constraints on the parameters, step by step (Meredith, 1993; Vandenberg and Lance, 2000). For a factor analysis model with non-zero means, the model is first specified by the following equation:

(1) $$\begin{array}{l} x=\tau_{x}+\Lambda_{x}\xi +\delta_{x} \end{array}$$

where, τ_*x*_ is defined as the intercept term for *x*, Λ_*x*_ is the factor loading term, ξ is the latent factor, and δ_*x*_ is the error of measurement related to *x*. Typical assumptions for δ* and ξ* are that *E*(δ) = 0 and *E*(ξ) = κ. Based on (1), the covariance structure can be derived from

(2) $$\begin{array}{l} \Sigma_{xx}=\Lambda_{x}\Phi \Lambda_{x}^{{}^{\prime }}+\Theta_{\delta_{x}} \end{array}$$

where Σ_*xx*_ is the sample variance–covariance matrix, which can be derived in terms of Λ_*x*_, Φ, and Θ_δ_*x*__. In addition, the mean structure can be identified through the following equation:

(3) $$\begin{array}{l} E\left(x\right)=\mu_{x}=\tau_{x}+\Lambda_{x}\kappa \end{array}$$

where κ is the mean of the latent factor. The mean of *x* can be viewed as a function of τ_*x*_, Λ_*x*_, and κ. Combined with the three equations, the full model for covariance and mean can be explained by the following parameters: Λ_*x*_, Φ, Θ_δ_*x*__, τ_*x*_, and κ. For detailed information, please refer to Meredith (1993) and Byrne et al. (1989). Therefore, MI for the CFA model could be evaluated *via* the following steps:

Step 1. An omnibus test of the equality of covariance matrices across groups. In other words, the null hypothesis, $H_{0}:\;\Sigma_{xx}^{\left(1\right)}=\;\Sigma_{xx}^{\left(2\right)}=\ldots =\Sigma_{xx}^{\left(g\right)}$, is tested in this step.

Step 2. To establish the configural invariance, a test to establish the presence of identical factorial patterns across groups.

Step 3. To confirm the metric invariance, a test for identical factor loadings of items across groups. That is to say, the null hypothesis, $H_{0}:\;\Lambda_{x}^{\left(1\right)}=\;\Lambda_{x}^{\left(2\right)}=\ldots =\Lambda_{x}^{\left(g\right)}$, is tested in this step.

Step 4. To demonstrate the scalar invariance, a test for equal intercepts of like items across groups. In other words, the null hypothesis, $H_{0}:\;\upsilon_{x}^{\left(1\right)}=\;\upsilon_{x}^{\left(2\right)}=\ldots =\upsilon_{x}^{\left(g\right)}$, is tested in this step.

Step 5. To establish uniqueness invariance, a test of whether the unique variances of the items are invariant across groups.

The tests below should proceed in order and only if the earlier test for equivalence has been passed.

Step 6. Test whether the factor variances are invariant across groups.

Step 7. Test whether the factor covariances are invariant across groups.

Step 8. Test whether the factor means are invariant across groups.

The MGCFA approach to invariance testing was deployed in this study. Additionally, we followed Byrne et al. (1989) by referring to the first five of these tests as tests of aspects of MI, since testing the relationships between the measured variables and latent constructs was one of the goals of the study. However, we excluded the test of invariant covariance matrices (Step 1), because this test is “uninformative with respect to the particular source of measurement inequivalence” (Vandenberg and Lance, 2000, p. 36).

### The Present Study

The study intended to examine the cross-national comparisons of science motivational beliefs of adolescents and gender differences within three countries, based on the most recent TIMSS data. However, to claim the validity of mean comparisons between groups, the MI of the constructs should first be assessed. To ensure the validity of the quantitative results (Meredith, 1993; Gaspard et al., 2017), this study also aimed to examine the degree of factor structure invariance of the science motivational beliefs of adolescents across countries and genders within countries. Although MI is a fundamental prerequisite to comparing means across groups, many studies have proceeded to make such comparisons without first establishing the existence of MI. To the best of our knowledge, no study has examined the MI of the three-factor science motivational beliefs based on the latest TIMSS data. It is our view that only when the MI has been established (i.e., research questions 1 and 3) should subsequent analyses regarding mean differences across groups be conducted (i.e., research questions 2 and 4). Accordingly, the research questions were framed as follows:

1. To what degree is the MI of science motivational beliefs achieved across the three countries?
2. What are the mean differences in science motivational beliefs across the three countries?
3. To what degree is the MI for science motivational beliefs by gender achieved in the three countries?
4. What are the gender differences in science motivational beliefs in the three countries?

## Materials and Methods

### Data

Data are from the portion of Taiwanese, Australian, and US eighth-grade students whose responses were measured in the most recent TIMSS in 2019. TIMSS employs a two-stage stratified sampling approach. First, schools are proportionately selected according to their size. The classrooms within the selected schools are subsequently chosen on a random basis. Further information regarding the data and sampling procedures of TIMSS can be found in the technical reports by Martin et al. (2020). Table 1 summarizes the sample size, percentage of sample size, and science achievement by gender in each country. It indicates that the science scores of males were non-significantly higher than those of females in Taiwan and Australia, whereas the scores of males were non-significantly lower than those of females in the United States. Responses with the missing values on all items were excluded from further analysis. Inspection of the individual item distributions showed the missing values were no more than 2.7% for any single item.

**Table 1:** Sample size, percentage of sample size, and science scores by gender in three countries.

**Country**	**Sample size**	**% of males**	**% of females**	**Male score (SE)**	**Female score (SE)**
Taiwan	4,915	50.50	49.50	576 (2.48)	572 (2.35)
Australia	9,060	50.50	49.50	529 (4.65)	528 (3.11)
United States	8,698	50.60	49.40	520 (6.07)	525 (3.88)

*SE, standard error*.

### Measures

The present study focused on three science motivational beliefs of adolescents, namely, self-concept in science, the intrinsic value of science, and its utility value of science. The section covering the self-concept of adolescents in science consists of eight items (i.e., SC1–SC8). An example item is “I usually do well in science.” The section investigating the beliefs about the intrinsic value of science is composed of nine items (i.e., IV1–IV9), such as “I enjoy learning science.” Finally, beliefs about the utility value of science are measured by nine items (i.e., UV1–UV9), such as “I think learning science will help me in my daily life.” All items were measured on a 4-point Likert scale ranging from 1 *(agree a lot)* to 4 *(disagree a lot)*. Negatively worded items were reverse scored. The reliability values of the scale scores for self-concept, intrinsic value, and utility value were 0.92, 0.92, and 0.93 for Taiwan, 0.89, 0.93, and 0.93 for Australia, and 0.87, 0.91, and 0.92, for the United States, respectively. The list of complete items for each science motivational belief is given in Appendix A.

### Analytic Strategy

Three sets of analytical approaches were utilized. First, CFA was used to evaluate the fit of the three-factor model for science motivational beliefs of adolescents. Second, the MI of the motivational beliefs across the three countries, as well as that of gender within each country, was tested. Third, the mean differences among the countries and genders were examined after MI was confirmed to have been achieved at the scalar level. Most of the analyses were conducted using *Mplus* 8.3 (Muthén and Muthén, 2017) with robust maximum likelihood estimation (MLR), which outperforms the conventional maximum likelihood (ML) for ordinal observed variables (Li, 2016). The default method for handling missing data was full information maximum likelihood, which was used with the MLR estimator based on the missing at random assumption for missing data. SAS software (SAS Institute, 2008) was utilized for data preprocessing. Due to the complex sampling of TIMSS, the effects of sampling weights and design effects were taken into account (Liou and Hung, 2015). Two variables, namely, student house weight (SENWGT) and student senate weight (HOUWGT), were used as the weighting variables to account for sampling of the school, class, and adolescents as well as adjustment factors corresponding to non-participation at the three levels. SENWGT was applied to the between-countries analyses, while HOUWGT was deployed for the within-country analyses. The variable IDCLASS was used to identify classes and was treated as a clustering variable to control for the cluster sample.

The procedures of each analytical approach are presented in the following paragraphs. First, CFA was performed to evaluate whether the three-factor measurement model reached an acceptable fit to the data in each country. The first loading for each factor was fixed at 1. The hypothesized model was used to fit the data. Furthermore, the extent to which the three-factor model fitted both females and males in each country was assessed. Subsequently, a series of MGCFA procedures was used to test the degree of MI for each country and gender.

**Figure 1:**
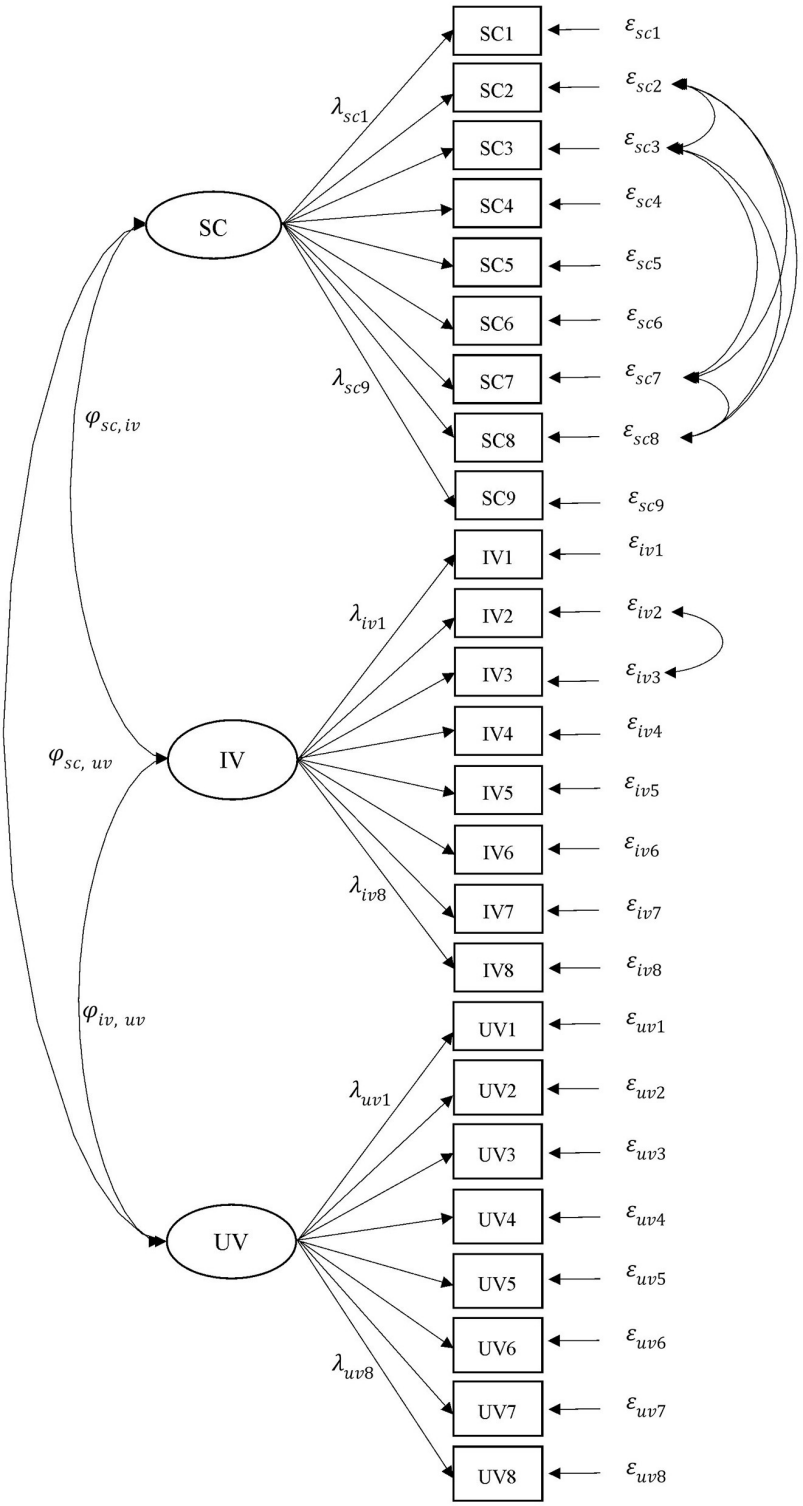
The measurement model for the three-factor model of science motivational beliefs. SC, self-concept; IV, intrinsic value; utility value; ϕ, correlation; λ, factor loading; ε, residual. The relations among residual terms (e.g., ε_*iv*2_↔ε_*iv*3_) were used to estimate the effects of the negative items. For simplicity, only the first (e.g., λ_*sc*1_) and the last (e.g., λ_*sc*9_) factor loadings for each factor are presented.

Four types of MI were tested, namely, MI across countries (MI-country), MI for gender in Taiwan (MI-Taiwan), MI for gender in Australia (MI-Australia), and MI for gender in the United States (MI-US). For each test of MI, six models (i.e., M1–M6) were developed, evaluated, and compared. M1 is a basic measurement model composed of three factors (i.e., SC, IV, and utility value). M2 is a model that incorporates the effects of negative wording into M1. This improvement draws on the study of Marsh et al. (2015a,b) who found the effects of the substantial method associated with negatively worded items when examining these motivational belief items in the TIMSS data. The present study accounted for this effect in the second model (M2). The negatively worded effect was controlled by correlated residuals for the observed items. Figure 1 illustrates the hypothesized measurement model (M1), in which a total of 26 items were used to estimate the three factors, as well as the negatively worded effects (M2) estimated in terms of relations among residuals (e.g., the relationship between ε_*iv*2_ and ε_*iv*3_).

Models M3–M6 were used to test the levels of MI. Starting with the least constrained model, M3 tests the configural invariance by constraining the factorial pattern to be identical for both females and males and freely estimating all parameters. M4 tests the metric invariance by fixing equal factor loadings for items across groups. For example, λ_*sc*9_ for females and λ_*sc*9_ for males are treated as equal. Previous researchers have suggested comparing cross-group variable means on latent variables after the scalar level of invariance has been confirmed (Hancock, 1997; Thompson and Green, 2013). Scalar invariance is the precondition for comparing latent factor means among groups (Marsh et al., 2009). M5 tests the scalar invariance by constraining the intercepts of like items to be equal. M6 tests the invariant uniqueness by constraining the unique variances of items as equal, for example, the variance of ε_*sc*2_ for females and the variance of ε_*sc*2_ are equalized. If the difference between these indices of fit falls beyond the acceptable values, testing for further MI should not be proceeded.

The indices of fit and criteria for checking the model fitting used in this study consist of the root mean square error of approximation (RMSEA; Browne and Cudeck, 1993), the comparative fit index (CFI; Bentler, 1990), Tucker–Lewis index (TLI; Tucker and Lewis, 1973), and the standardized root mean square residual (SRMR; Hu and Bentler, 1999). Regarding the cutoff values, an RMSEA value of <0.05 suggests a good fit, while the value of <0.08 reflects a minimal acceptable fit (Hu and Bentler, 1999). A CFI value >0.90 or 0.95 indicates that models are either an acceptable fit or an excellent fit to the data, respectively (Hu and Bentler, 1999). Bentler and Bonett (1980) suggested that a TLI value >0.90 indicates an acceptable fit, while an SRMR value of <0.08 indicates a minimal acceptable fit (Hu and Bentler, 1999).

To develop a more parsimonious model, comparisons based on various indices were conducted for models M3–M6. First, a decrease in the CFI value of <0.01 (Cheung and Rensvold, 2002) and <0.015 for RMSEA (Chen, 2007) were established as criteria for meeting each test of MI. Such rules are consistent with the recent research of Marsh et al. (2018), which focused on the issue of MI in complex surveys (e.g., TIMSS). In addition, ΔCFI was included as a particularly promising fit index for the evaluation of MI (Cheung and Rensvold, 2002). The results of this study are comparable to those of the aforementioned studies. To boost the validity, a variety of indices was deployed, following the recommendations of Marsh and Hau (2004). Therefore, the Gamma-Hat index (GFI; Steiger, 1998) and McDonald's Non-Centrality index (NCI; McDonald, 1989) were also reported as a supplemental reference. The cutoff value for GFI is 0.001, which means a parsimonious model is favorable, given the decrease in the GFI value to <0.001 (Chen, 2007). Likewise, the cutoff value for NCI is 0.01 (Kang et al., 2016). As *Mplus* does not provide the GFI and NCI values directly, an SAS procedure was developed to calculate the three indices based on the *Mplus* outputs.

Mean comparisons across counties and genders within countries were made after the scalar invariance was confirmed, following Steinmetz et al. (2009). In the SEM framework, group differences were compared in terms of the latent factor means of each latent construct (Sass, 2011). The basic idea was to constrain the latent factor mean of one group (the default group) to zero and to assess the mean difference between this and the other group. The difference was then tested with *t* statistics.

## Results

### Statistical Analysis of Science Motivational Beliefs

The gender-specific descriptive analysis and correlation of each item of the three science motivational beliefs for gender within each country were conducted. For females, the average scores for self-concept ranged from *M* = 2.01–3.33 [standard deviation (*SD*) = 0.74–1.03], and from *M* = 2.32–3.35 (*SD* = 0.78–1.02) for males. The correlations between the self-concept items ranged from *r* = 0.26–0.75 (females) and *r* = 0.13–0.80 (males). In terms of intrinsic value, the average scores varied from *M* = 2.34–3.40 (*SD* = 0.72–1.07) for females and from *M* = 2.68–3.48 (*SD* = 0.77–1.05) for males. The correlations between the items of the intrinsic value ranged from *r* = 0.29–0.84 for females and *r* = 0.21–0.83 for males. In terms of the utility value, the average scores ranged from *M* = 2.14–3.47 (*SD* = 0.77–1.10) for females and from *M* = 2.48–3.39 (*SD* = 0.81–1.07) for males. The correlations between utility value items ranged from *r* = 0.34–0.86 for females and *r* = 0.40–0.81 for males. The country-specific mean values and correlations are given in Appendices B,C.

### Measurement Inequivalence of Science Motivational Beliefs Across Countries

The results indicated that the MI of the science motivational beliefs of adolescents across the three countries attained only the metric invariance. The indices for the data presented in Table 2 indicate an improved fit after the effects of negatively worded items are taken into account. For the first model (M1), the results suggested that M1 fit the data poorly, χ^2^ (296, *N* = 22,014) = 21,169.49, RMSEA = 0.057, CFI = 0.720, TLI = 0.693, and SRMR = 0.063. However, when the effects of negative wording were considered (M2), the goodness of fit improved, χ^2^ (289, *N* = 22,014) = 10,280.96, RMSEA = 0.040, CFI = 0.866, TLI = 0.849, and SRMR = 0.067. These results confirm the likely effects of negative wording on the responses to the items. The results of M3 indicated the configural invariance, χ^2^ (867, *N* =22,014) = 23,383.68, RMSEA = 0.059, CFI = 0.927, TLI = 0.918, and SRMR = 0.050. In other words, the factorial constructs were equal across countries. We subsequently examined the metric invariance (M4) by constraining the factor loadings of each country to be equal. The testing difference of the fit indices supported the invariance of factor loadings across the three countries (ΔRMSEA <0.001, ΔCFI = 0.004). In other words, the relationship of each item to the underlying factor was equal across countries. Scalar invariance was examined by constraining the intercepts for each item at equal levels across grades, with the results indicating the absence of this form of invariance (ΔRMSEA = 0.004, ΔCFI = 0.015).

**Table 2:** The goodness-of-fit statistics for the nested CFA models across countries and genders.

**Model**	**χ^**2**^**	***df***	**RMSEA**	**CFI**	**TLI**	**SRMR**
**MI country**						
M1	21169.49	296	0.057	0.720	0.693	0.063
M2	10280.96	289	0.040	0.866	0.849	0.047
M3	23383.68	867	0.059	0.927	0.918	0.050
M4	24439.55	913	0.059	0.923	0.918	0.058
M5	29204.37	959	0.063	0.908	0.907	0.069
**MI Taiwan**						
M1	10210.46	296	0.083	0.843	0.827	0.071
M2	5083.11	289	0.058	0.924	0.914	0.058
M3	6079.40	578	0.062	0.925	0.916	0.059
M4	6340.13	601	0.062	0.922	0.915	0.069
M5	6649.48	624	0.063	0.918	0.914	0.070
M6	6768.16	657	0.062	0.917	0.917	0.073
**MI Australia**						
M1	09654.11	296	0.060	0.873	0.860	0.060
M2	05788.99	289	0.046	0.925	0.916	0.046
M3	10412.06	578	0.062	0.925	0.915	0.047
M4	10698.53	601	0.062	0.923	0.916	0.052
M5	11127.01	624	0.062	0.920	0.916	0.053
M6	11298.05	657	0.061	0.919	0.919	0.056
**MI US**						
M1	11908.04	296	0.069	0.806	0.787	0.070
M2	5526.68	289	0.047	0.912	0.901	0.052
M3	8651.07	578	0.058	0.923	0.913	0.053
M4	8910.90	601	0.058	0.920	0.914	0.060
M5	9350.25	624	0.058	0.916	0.913	0.061
M6	9439.75	657	0.057	0.916	0.917	0.063

*MI, measurement invariance; M1, confirmatory factor analysis; M2, confirmatory factor analysis with a consideration of the negative effects; M3, configural invariance; M4, metric invariance; M5, scalar invariance; M6, residual invariance; RMSEA, root mean square error of approximation; CFI, comparative fit index; TLI, Tucker–Lewis index; SRMR, standardized root mean square residual*.

### Measurement Equivalence for Science Motivational Beliefs by Gender Within Countries

Table 3 presents the levels of MI examined based on a comparison of the fit indices of the nested models. The results suggested that the MI of science motivation of adolescents reached invariance uniqueness in each of the three countries. By the way of illustration, the results of adolescents in Taiwan are presented below, with the MI results for adolescents in Australia and the United States documented in Appendix D.

**Table 3:** Model comparisons for the nested models.

**Model**	**χ^**2**^**	***df***	**$\chi_{\text{diff}}^{\text{2}}$**	**Δ*df***	**RMSEA (90% CI)**	**ΔRMSEA**	**CFI**	**ΔCFI**	**NCI**	**ΔNCI**	**GFI**	**ΔGFI**
**MI-Country**												
M3	23383.68	867	–	–	0.059 (0.058–0.059)	–	0.927	–	0.600	–	0.998	–
M4	24439.55	913	1055.87***	46	0.059 (0.058–0.059)	<0.001	0.923	0.004	0.586	0.014	0.998	<0.001
M5	29204.37	959	4764.82***	46	0.063 (0.064–0.065)	0.004	0.908	0.015	0.526	0.060	0.997	<0.001
**MI-Taiwan**												
M3	06079.40	578	–	–	0.062 (0.061–0.064)	–	0.925	–	0.571	–	0.997	–
M4	06340.13	601	260.73***	23	0.062 (0.061–0.064)	<0.001	0.922	0.003	0.558	0.014	0.996	<0.001
M5	06649.48	624	309.35***	23	0.063 (0.062–0.065)	0.001	0.918	0.004	0.542	0.016	0.996	<0.001
M6	06768.16	657	118.68***	33	0.062 (0.060–0.063)	<0.001	0.917	0.001	0.537	0.005	0.996	<0.001
**MI Australia**												
M3	10412.06	578	–	–	0.062 (0.061–0.063)	–	0.925	–	0.574	–	0.997	–
M4	10698.53	601	286.47***	23	0.062 (0.061–0.063)	0	0.923	0.002	0.565	0.008	0.997	<0.001
M5	11127.01	624	428.48***	23	0.062 (0.061–0.063)	0	0.920	0.003	0.552	0.013	0.996	<0.001
M6	11298.05	657	171.04***	33	0.061 (0.060–0.061)	<0.001	0.919	0.001	0.548	0.004	0.996	<0.001
**MI-USA**												
M3	08651.07	578	–	–	0.058 (0.057–0.059)	–	0.923	–	0.613	–	0.997	–
M4	08910.90	601	259.83***	23	0.058 (0.057–0.059)	0	0.920	0.003	0.604	0.009	0.997	<0.001
M5	09350.25	624	439.35***	23	0.058 (0.057–0.060)	0	0.916	0.004	0.589	0.015	0.997	<0.001
M6	09439.75	657	89.50***	33	0.057 (0.056–0.058)	<0.001	0.916	0.000	0.587	0.002	0.997	<0.001

*MI, measurement invariance; M1, confirmatory factor analysis; M2, confirmatory factor analysis with a consideration of the negative effects; M3, configural invariance; M4, metric invariance; M5, scalar invariance; M6, residual invariance*.

$\chi_{\text{diff}}^{\text{2}}$, nested χ^2^ difference; RMSEA, root mean square error of approximation; CI, confidence interval; CFI, comparative fit index; NCI, McDonald's Non-Centrality index; GFI, Gamma-Hat index. ***p < 0.001.

The MI of the science motivational beliefs of Taiwanese adolescents attained invariant uniqueness. The fit indices for M1 showed a poor fit, χ^2^ (296, *N* = 4,913) = 10,210.46, RMSEA = 0.083, CFI = 0.843, TLI = 0.827, and SRMR = 0.071. The indices of M2 were improved after accounting for the effect of negative wording, χ^2^ (289, *N* = 4,913) = 5,083.11, RMSEA = 0.058, CFI = 0.924, TLI = 0.914, and SRMR = 0.058. These results indicate strong support for the three-factor model. Moreover, they suggest that the negatively worded items substantially impacted the fit of the model. The results of M3 support the finding that the factorial construct was equal across genders, χ^2^ (578, *N* = 4,913) = 6,079.40, RMSEA = 0.062, CFI = 0.925, TLI = 0.916, and SRMR = 0.059. Subsequently, metric invariance (M4) was examined by constraining the factor loading of males and females to be equal. The testing difference of the fit indices indicated that the factor loadings were equal across groups (ΔRMSEA = 0, ΔCFI = 0.003). In other words, the relationship of each item to the underlying factor was equal across both the genders. The investigation into scalar invariance (M5) was accomplished by constraining the intercepts for each item to be equal across groups, with the results indicating that the scalar invariance had indeed been achieved (ΔRMSEA = 0.001, ΔCFI = 0.004). In other words, the intercepts of like items were identical across gender. Finally, the presence of uniqueness invariance (M6) was examined by constraining the unique variances for each item to be equal across groups. The results revealed the existence of unique invariance in the items (ΔRMSEA < 0.001, ΔCFI = 0.001), each of which achieved the uniqueness invariance across gender.

Table 4 presents the estimated standardized covariance among student responses regarding science self-concept, its intrinsic value, and its utility value. All factor loadings were significant. The standardized covariance for females ranged from 0.52 to 0.79 for females and from 0.58 to 0.81 for males. These high values among the three factors pointed to the likelihood of a higher-order general factor in the three science motivational beliefs. In addition, the standardized covariance for males was higher than that for females.

**Table 4:** Estimated standardized covariance among science motivational beliefs.

	**Self-concept**	**Intrinsic value**	**Utility value**
Self-concept	–	Taiwan: 0.76 Australia: 0.81 United States: 0.76	Taiwan: 0.66 Australia: 0.63 United States: 0.58
Intrinsic value	Taiwan: 0.77 Australia: 0.79 United States: 0.75	–	Taiwan: 0.68 Australia: 0.69 United States: 0.65
Utility value	Taiwan: 0.62 Australia: 0.55 United States: 0.52	Taiwan: 0.67 Australia: 0.64 United States: 0.61	–

*Lower triangle: female and upper triangle: male*.

### Gender Differences in Science Motivational Beliefs

As mentioned in the Analytic Strategy section, comparison of groups can occur only after the scalar invariance has been confirmed. However, the MI results suggested that the scalar invariance across countries was absent (ΔCFI > 0.01), and thus, the factor means for Taiwan, Australia, and the United States were not meaningfully comparable.

However, the mean differences between females and males on the three latent science motivational beliefs in each country were examined. Table 5 presents these differences and the corresponding statistics for the three sets of science motivational beliefs in Taiwan, Australia, and the United States. The results suggested that males attained higher scores on the three constructs (i.e., self-concept, intrinsic value, and utility value) than females. The pattern was consistent across the three countries, except for the insignificant difference between females and males for utility value in the United States.

**Table 5:** Comparisons of latent means of factors between females and males in the three countries.

**Country**	**Factor**	***M* (*SE*)**	**diff_**Gender**_**	***t***	***p***
Taiwan	Self-concept	0.32 (0.02)	0.32	13.37	<0.01
	Intrinsic value	0.25 (0.02)	0.25	10.45	<0.01
	Utility value	0.14 (0.02)	0.13	7.85	<0.01
Australia	Self-concept	0.20 (0.02)	0.20	11.42	<0.01
	Intrinsic value	0.16 (0.02)	0.16	7.01	<0.01
	Utility value	0.07 (0.02)	0.07	3.74	<0.01
United States	Self-concept	0.14 (0.02)	0.13	9.11	<0.01
	Intrinsic value	0.12 (0.02)	0.12	6.12	<0.01
	Utility value	−0.01 (0.02)	−0.01	−0.60	0.55

*M, mean; SE, standard error. Given that the reference group was female, the mean and corresponding SE refer to the average factor score and the standard error of the male group. diff_Gender_, factor mean of male values – factor mean of female values*.

## Discussion

Comparisons of the science motivational beliefs of adolescents across nations and by gender are important research topics. Numerous studies have examined earlier such phenomena using TIMSS data. However, failing to establish equivalency prior to group comparisons “could be threatening to substantive interpretations as is an inability to demonstrate reliability and validity” (Vandenberg and Lance, 2000, p. 6). To this end, the present study aimed to investigate the MI of three key science motivational beliefs among adolescents across three countries (Taiwan, Australia, and the United States) and by gender within each country. This study expands our understanding of the psychometric characteristics of the science motivational beliefs of adolescents across groups and of the substantive educational issue of gender disparities in this domain. The following sections present the MI of the science motivation beliefs of adolescents by gender, the gender disparities in such beliefs in the three countries based on valid constructs, and indefensible measures of science motivational beliefs of adolescents for cross-national comparisons. At the end of this article, the limitations and potential directions for future research are suggested.

### Well-Evaluated Psychometric Properties of Science Motivational Beliefs for the Comparison of Gender Differences

The validity of conclusions about mean gender differences in science motivational beliefs of adolescents is dependent on scales that are themselves valid and reliable. The minimum prerequisite for meaningful examination of mean differences is scalar invariance of the relevant measure. Otherwise, we simply compared the apples with oranges, i.e., the survey data will be non-comparable (Greiff and Scherer, 2018). However, while confirming the MI of constructs to ensure a valid comparison across groups is essential, it is not easily achieved. For instance, the study of Frenzel et al. (2012) failed to establish the MI for research suggesting that intrinsic interest of students in mathematics was more significant than grade-making, whereas many earlier studies (e.g., Liou and Liu, 2015; Ghasemi and Burley, 2019; Mejía-Rodríguez et al., 2020), which directly used the average scale scores of psychological constructs of students, overlooked the importance of MI entirely.

To remedy the deficiencies in extant studies, one major contribution of this research has been to demonstrate the detailed statistical procedures required to provide empirical evidence for further quantitative studies of gender disparities, with a particular focus on comparing the science motivational beliefs of adolescents. First, the three-factor structure for these beliefs was strongly supported. Furthermore, a series of MI of the beliefs in Taiwan, Australia, and the United States was verified according to gender. While the process of checking the MI of the beliefs of adolescents drew on established procedures, the method of analyzing the effects of negatively worded items was a particular contribution of this study. Consistent with the earlier studies (e.g., Marsh et al., 2015a,b), the fit indices for the models indicated that the inclusion of correlating residuals of the negatively worded items substantially improved the goodness of fit. This should generate further discussion and development of rigorous analyses of survey items with both positive and negative items.

### Concerns Regarding Measurement Equivalency of Science Motivational Beliefs for International Comparisons

Measurement equivalency is an important prerequisite for the subsequent comparison of group differences in construct scores. However, the findings of this study showed that MI did not hold for the comparisons across the three countries. This implies that adolescents from Taiwan, Australia, and the United States interpreted the items and scales of science motivational beliefs in ways that were not identical. In other words, if adolescents of the same science motivational belief trait scored differently on the TIMSS items, this was due to some other background variables, such as the country and culture, in this case. Future studies are needed for the national and cultural differences on forming adolescent science motivational beliefs.

While TIMSS is widely known for the international reach of its data, the results of this study question the MI of constructs across countries and the resulting comparisons across countries. Many cross-country studies based on TIMSS data have been conducted; however, it has been shown that cultural bias may emerge when comparing the perceptions of the students, such as motivational beliefs. As earlier research has shown, it is difficult to attain the scalar and even residual invariance of the constructs when testing cross-cultural patterns of adolescent motivational beliefs (Marsh et al., 2013, 2015a,b; Asparouhov and Muthén, 2014). There is a very real risk that further comparisons between countries may be invalid. Researchers faced with such an absence of evidence of the MI issue may narrow their focus to individual countries rather than making trans-national comparisons in areas such as science motivational beliefs.

### Gender Disparities in Science Motivational Beliefs Across Countries

The self-concept of adolescent males in science and their beliefs about its intrinsic value were statistically higher than those of females in all three countries. As for the utility value of science, adolescents in Taiwan and Australia showed the same pattern as the other two science motivational beliefs, but there was no statistical difference for adolescents in the United States. These results corroborated the majority of studies (e.g., Marsh et al., 2013; Wang and Degol, 2017), which have focused on gender disparities in science motivational beliefs. Among the three motivational beliefs, the gender gap around the utility value of science was the narrowest and was not significant in the United States. Compared to the self-concept and the intrinsic value, the utility value is recognized as being the most sensitive to interventions and external manipulation (Hulleman et al., 2010; Rozek et al., 2015). The result of this study signals the need for further research on closing the gender gap for the other two science motivational beliefs and for the self-concept of adolescents especially in science.

The gender gap in science motivational beliefs was most pronounced among adolescents in Taiwan. Given the absence of such a difference for science cognitive achievement (Mullis et al., 2020), this finding appears paradoxical. We speculated that the influence of gender-stereotypical socialization is more powerful in Taiwan than in Australia and the United States. Denissen et al. (2007) described the dissimilarity in academic motivational beliefs as a gender-stereotypical difference: science is often considered a male-dominated field. As adolescence is the key period where the beliefs of students influence their choice of future majors and domains of employment, a wide gap in science self-concept at this stage may prevent female students from pursuing careers in STEM fields. Since the formation of the self-concept of adolescents is impacted through schooling and socialization, how best to utilize these processes to promote the self-concept in females in science remains a topic for further extensive discussion.

### Limitations and Directions for Future Research

The limitations of this study and associated recommendations for future research will be discussed in this section. First, it is important to further examine the correlations between the science motivational beliefs of students and other educational outcomes, course selection, and even career choices in STEM fields. By addressing the methodological deficiencies of much earlier research, the present study established the MI of science motivational beliefs in Taiwan, Australia, and the United States. As a result, we provided the first profound evidence for the validity of comparative results on gender disparities in science motivational beliefs and actual educational outcomes for the three countries.

Second, following the above suggestion, future studies are encouraged to investigate whether the results can be generalized to other countries. The issues raised can also be studied within the context of a single country: such research will be able to draw upon the demonstration of the present study of MI in science motivational beliefs across genders. However, as this study demonstrated the lack of MI of science motivational beliefs across the three countries in the TIMSS data, researchers who want to use all three countries for comparative purposes should engage in scalar refinement that would allow for measurement equivalency.

Finally, while the science motivational beliefs of students are the focus of this study, other constructs regarding learning science and mathematics can also be extracted from TIMSS, which provides rich information regarding the backgrounds and achievement of the students—not only in science but also in mathematics. This will advance our understanding of the correlations between various factors in STEM education and how they influence the teaching and learning process.

## Data Availability Statement

The raw data supporting the conclusions of this article can be downloaded from the TIMSS website (https://timss2019.org/international-database/#).

## Ethics Statement

Ethical review and approval was not required for the study on human participants in accordance with the local legislation and institutional requirements.

## Author Contributions

P-YL mainly contributes to the writing of the introduction, literature review, method, and discussion sections. JL contributes to the writing of methods and results sections and responsible for the statistical design and analysis. All authors contributed to the article and approved the submitted version.

## Conflict of Interest

The authors declare that the research was conducted in the absence of any commercial or financial relationships that could be construed as a potential conflict of interest.

## Publisher's Note

All claims expressed in this article are solely those of the authors and do not necessarily represent those of their affiliated organizations, or those of the publisher, the editors and the reviewers. Any product that may be evaluated in this article, or claim that may be made by its manufacturer, is not guaranteed or endorsed by the publisher.

## Footnotes

^1^Gender refers to the biological sex to stay consistent with the earlier STEM research.
