# Supplementary material for: Comparisons of Science Motivational Beliefs of Adolescents in Taiwan, Australia, and the United States: Assessing the Measurement Invariance Across Countries and Genders
Source: Front Psychol. 2021 Aug 2;12:674902. doi: 10.3389/fpsyg.2021.674902 (PMC8365248; doi:10.3389/fpsyg.2021.674902)
Supplement: Supplementary file 1 [file Table_1.pdf]

Liou, P.-Y., & Lin, J. J. H. (2021). Comparisons of science motivational beliefs of adolescents in Taiwan, Australia, and the United States: Assessing the measurement invariance across countries and genders. *Frontiers in Psychology.* doi: 10.3389/fpsyg.2021.674902

Supplementary Materials

Appendix A. Item Descriptions for Adolescents' Science Motivational Beliefs

Self-concept in Science	
I usually do well in science.	SC1
Science is more difficult for me than for many of my classmates.	SC2
Science is not one of my strengths.	SC3
I learn things quickly in science.	SC4
I am good at working out difficult science problems.	SC5
My teacher tells me I am good at science.	SC6
Science is harder for me than any other subject.	SC7
Science makes me confused.	SC8
Intrinsic Value of Science	
I enjoy learning science.	IV1
I wish I did not have to study science.	IV2
Science is boring.	IV3
I learn many interesting things in science.	IV4
I like science.	IV5
I look forward to learning science in school.	IV6
Science teaches me how things in the world work.	IV7
I like to conduct science experiments.	IV8
Science is one of my favorite subjects.	IV9
Utility Value of Science	
I think learning science will help me in my daily life.	UV1
I need science to learn other school subjects.	UV2
I need to do well in science to get into the university of my choice.	UV3
I need to do well in science to get the job I want.	UV4
I would like a job that involves using science.	UV5
It is important to learn about science to get ahead in the world.	UV6
Learning science will give me more job opportunities when I am an adult.	UV7
My parents think that it is important that I do well in science.	UV8
It is important to do well in science.	UV9

Appendix B. (a) Descriptive Statistics for Items Measuring Science Motivational Beliefs in Taiwan, Australia, and the United States (Females)

	Taiwan				Australia				United States			
Item	*M*	*SD*	Skewness	Kurtosis	*M*	*SD*	Skewness	Kurtosis	*M*	*SD*	Skewness	Kurtosis
SC1	2.42	0.81	0.15	-0.45	2.97	0.83	-0.58	-0.07	3.33	0.79	-1.09	0.74
SC2	2.41	0.91	0.08	-0.79	2.84	0.89	-0.36	-0.62	2.97	0.95	-0.54	-0.71
SC3	2.22	0.91	0.34	-0.68	2.52	0.99	-0.02	-1.02	2.73	1.02	-0.26	-1.08
SC4	2.21	0.74	0.45	0.16	2.69	0.86	-0.10	-0.68	3.03	0.89	-0.54	-0.58
SC5	2.06	0.74	0.55	0.40	2.44	0.87	0.07	-0.67	2.83	0.94	-0.31	-0.87
SC6	2.01	0.75	0.52	0.17	2.43	0.93	0.03	-0.86	2.71	1.03	-0.25	-1.09
SC7	2.34	0.92	0.14	-0.84	2.88	0.93	-0.45	-0.68	3.03	0.98	-0.68	-0.61
SC8	2.41	0.92	0.06	-0.85	2.56	0.97	-0.01	-0.98	2.75	1.01	-0.23	-1.08
IV1	2.68	0.80	-0.14	-0.43	2.94	0.92	-0.61	-0.42	3.11	0.91	-0.84	-0.06
IV2	2.67	0.86	-0.25	-0.53	2.77	0.99	-0.30	-0.95	2.70	1.02	-0.20	-1.09
IV3	2.77	0.82	-0.38	-0.27	2.69	0.97	-0.20	-0.96	2.76	1.00	-0.26	-1.04
IV4	2.97	0.74	-0.54	0.30	3.21	0.83	-0.90	0.25	3.30	0.83	-1.09	0.60
IV5	2.61	0.84	-0.01	-0.62	2.88	0.97	-0.50	-0.74	3.08	0.94	-0.78	-0.33
IV6	2.50	0.84	0.17	-0.59	2.67	0.99	-0.18	-1.00	2.92	0.98	-0.47	-0.84
IV7	3.01	0.72	-0.58	0.53	3.26	0.80	-0.99	0.63	3.30	0.83	-1.09	0.60
IV8	3.07	0.78	-0.60	0.03	3.40	0.79	-1.31	1.20	3.34	0.86	-1.22	0.67
IV9	2.34	0.86	0.35	-0.47	2.45	1.06	0.09	-1.22	2.79	1.07	-0.36	-1.15
UV1	2.99	0.77	-0.56	0.14	3.00	0.89	-0.61	-0.36	3.08	0.92	-0.73	-0.34
UV2	2.39	0.82	0.33	-0.39	2.71	0.93	-0.18	-0.85	2.81	0.98	-0.30	-0.98
UV3	2.54	0.91	0.10	-0.84	2.89	1.01	-0.44	-0.95	3.18	0.93	-0.88	-0.25
UV4	2.35	0.88	0.34	-0.56	2.81	1.06	-0.32	-1.16	2.95	1.03	-0.51	-0.99

UV5	2.14	0.84	0.62	0.02	2.54	1.08	-0.01	-1.28	2.70	1.10	-0.21	-1.29
UV6	2.42	0.86	0.15	-0.62	3.02	0.90	-0.62	-0.44	3.13	0.91	-0.77	-0.32
UV7	2.52	0.89	0.03	-0.74	3.12	0.91	-0.78	-0.28	3.20	0.91	-0.90	-0.15
UV8	2.63	0.92	-0.06	-0.86	3.20	0.84	-0.82	-0.03	3.34	0.84	-1.14	0.51
UV9	2.84	0.86	-0.41	-0.42	3.30	0.79	-1.01	0.61	3.43	0.79	-1.38	1.37

Note. SC = self-concept in science; IV = intrinsic value of science; UV = utility value.

Appendix B. (a) Descriptive Statistics for Items Measuring Science Motivational Beliefs in Taiwan, Australia, and the United States (Males)

	Taiwan				Australia				United States			
Item	*M*	*SD*	Skewness	Kurtosis	*M*	*SD*	Skewness	Kurtosis	*M*	*SD*	Skewness	Kurtosis
SC1												
SC2	2.69	0.89	-0.16	-0.74	3.10	0.81	-0.72	0.14	3.35	0.78	-1.17	0.99
SC3	2.62	0.97	-0.14	-0.96	2.89	0.92	-0.41	-0.71	2.99	0.95	-0.57	-0.69
SC4	2.52	1.00	-0.02	-1.07	2.71	0.99	-0.20	-1.04	2.79	1.02	-0.32	-1.07
SC5	2.56	0.89	0.07	-0.78	2.92	0.86	-0.39	-0.57	3.13	0.87	-0.70	-0.32
SC6	2.40	0.90	0.23	-0.71	2.74	0.89	-0.19	-0.75	2.97	0.92	-0.50	-0.66
SC7	2.32	0.89	0.31	-0.59	2.63	0.95	-0.18	-0.88	2.79	1.02	-0.36	-1.00
SC8	2.59	0.99	-0.12	-1.01	2.99	0.94	-0.60	-0.57	3.05	0.98	-0.72	-0.58
IV1	2.67	0.99	-0.20	-0.99	2.76	0.96	-0.21	-0.98	2.81	1.01	-0.31	-1.04
IV2	2.96	0.88	-0.52	-0.43	3.11	0.92	-0.86	-0.08	3.18	0.90	-0.98	0.21
IV3	2.84	0.95	-0.45	-0.70	2.81	1.03	-0.36	-1.05	2.68	1.04	-0.19	-1.15
IV4	2.93	0.90	-0.56	-0.41	2.81	1.00	-0.34	-0.99	2.78	1.02	-0.30	-1.06
IV5	3.08	0.83	-0.72	0.09	3.27	0.85	-1.07	0.50	3.33	0.84	-1.19	0.82
IV6	2.91	0.91	-0.43	-0.68	3.06	0.94	-0.76	-0.32	3.16	0.93	-0.90	-0.11
IV7	2.73	0.94	-0.18	-0.91	2.87	0.98	-0.46	-0.82	2.99	0.96	-0.58	-0.70
IV8	3.11	0.82	-0.82	0.35	3.30	0.82	-1.11	0.76	3.32	0.82	-1.14	0.75
IV9	3.19	0.85	-0.92	0.26	3.48	0.77	-1.54	1.97	3.34	0.88	-1.23	0.67
UV1	2.69	0.96	-0.12	-0.99	2.75	1.05	-0.29	-1.14	2.92	1.04	-0.55	-0.91
UV2	3.11	0.84	-0.81	0.21	3.09	0.88	-0.72	-0.23	3.09	0.94	-0.78	-0.32
UV3	2.55	0.93	0.10	-0.88	2.85	0.93	-0.30	-0.87	2.86	0.99	-0.39	-0.94
UV4	2.70	0.96	-0.12	-0.99	2.99	0.97	-0.58	-0.74	3.17	0.93	-0.87	-0.23

UV5	2.56	0.94	0.08	-0.94	2.90	1.01	-0.43	-0.97	2.93	1.03	-0.50	-0.97
UV6	2.48	0.95	0.18	-0.92	2.70	1.05	-0.17	-1.19	2.74	1.07	-0.26	-1.20
UV7	2.63	0.93	-0.10	-0.88	3.09	0.90	-0.72	-0.33	3.12	0.92	-0.79	-0.28
UV8	2.70	0.93	-0.16	-0.87	3.20	0.89	-0.90	-0.03	3.20	0.91	-0.93	-0.05
UV9	2.70	0.94	-0.17	-0.91	3.23	0.84	-0.87	-0.01	3.32	0.84	-1.13	0.53

Note. SC = self-concept in science; IV = intrinsic value of science; UV = utility value.

Appendix C. (a) Correlation among Observed Items of the Three Science Motivational Beliefs in Taiwan

	SC1	SC2	SC3	SC4	SC5	SC6	SC7	SC8	IV1	IV2	IV3	IV4	IV5	IV6	IV7	IV8	IV9	UV1	UV2	UV3	UV4	UV5	UV6	UV7	UV8	UV9
SC1	-	.48	.53	.78	.73	.72	.43	.42	.63	.36	.37	.52	.61	.57	.50	.43	.65	.49	.46	.51	.47	.52	.43	.47	.39	.49
SC2	.59	-	.76	.45	.42	.40	.67	.60	.38	.45	.43	.30	.39	.34	.26	.21	.40	.23	.20	.28	.22	.28	.18	.22	.16	.24
SC3	.62	.75	-	.50	.46	.44	.69	.64	.43	.45	.46	.35	.44	.40	.30	.26	.48	.27	.24	.29	.25	.33	.21	.26	.17	.27
SC4	.69	.53	.58	-	.80	.72	.44	.42	.63	.36	.37	.53	.63	.60	.50	.43	.68	.51	.49	.53	.48	.56	.45	.48	.37	.49
SC5	.65	.48	.52	.74	-	.76	.38	.37	.56	.32	.32	.46	.56	.56	.44	.38	.62	.45	.48	.50	.49	.56	.44	.46	.37	.44
SC6	.67	.51	.54	.67	.70	-	.35	.34	.51	.29	.29	.42	.52	.51	.41	.34	.56	.42	.47	.49	.47	.51	.41	.45	.39	.44
SC7	.55	.63	.67	.55	.50	.47	-	.76	.37	.44	.44	.30	.39	.35	.24	.22	.42	.20	.16	.22	.18	.27	.16	.20	.10	.19
SC8	.54	.60	.63	.53	.47	.45	.75	-	.40	.46	.47	.32	.43	.38	.27	.22	.42	.24	.19	.22	.19	.26	.16	.20	.10	.22
IV1	.61	.43	.47	.60	.53	.47	.50	.50	-	.45	.46	.70	.82	.75	.65	.58	.77	.55	.47	.49	.45	.53	.43	.46	.32	.50
IV2	.44	.41	.46	.42	.39	.36	.46	.48	.55	-	.67	.37	.46	.42	.32	.26	.44	.33	.25	.27	.24	.31	.23	.27	.13	.28
IV3	.44	.41	.44	.42	.37	.33	.45	.48	.57	.68	-	.42	.49	.45	.37	.30	.47	.36	.24	.27	.24	.30	.23	.26	.12	.30
IV4	.46	.31	.33	.46	.40	.35	.35	.35	.64	.45	.52	-	.75	.69	.70	.61	.68	.54	.43	.46	.41	.45	.41	.45	.31	.49
IV5	.60	.42	.47	.60	.55	.49	.51	.50	.80	.56	.56	.68	-	.80	.70	.61	.82	.57	.48	.51	.47	.54	.45	.49	.34	.53
IV6	.53	.35	.40	.55	.50	.44	.44	.45	.73	.52	.51	.63	.78	-	.66	.58	.79	.52	.49	.49	.47	.54	.47	.48	.34	.51
IV7	.40	.24	.25	.39	.36	.30	.26	.27	.50	.33	.34	.57	.52	.53	-	.64	.65	.60	.43	.48	.43	.45	.45	.48	.37	.54
IV8	.35	.21	.22	.35	.30	.25	.23	.25	.48	.31	.35	.54	.49	.48	.48	-	.61	.46	.36	.40	.36	.40	.38	.39	.30	.46
IV9	.63	.44	.53	.64	.59	.55	.53	.51	.74	.53	.53	.60	.77	.72	.50	.48	-	.53	.49	.52	.51	.59	.47	.50	.36	.53
UV1	.44	.29	.30	.43	.38	.35	.32	.33	.52	.42	.40	.53	.51	.48	.53	.39	.49	-	.60	.58	.54	.54	.54	.57	.45	.65
UV2	.39	.27	.28	.41	.41	.37	.29	.30	.45	.35	.33	.41	.46	.46	.38	.31	.47	.54	-	.65	.65	.65	.59	.61	.51	.54
UV3	.41	.28	.31	.41	.37	.39	.25	.27	.43	.32	.32	.37	.44	.40	.39	.27	.44	.50	.56	-	.81	.70	.67	.73	.59	.65
UV4	.39	.26	.28	.43	.41	.40	.25	.28	.44	.33	.31	.36	.45	.42	.33	.26	.46	.46	.58	.76	-	.78	.71	.76	.57	.63
UV5	.46	.33	.37	.52	.51	.48	.34	.36	.52	.41	.37	.41	.52	.50	.31	.30	.56	.44	.55	.59	.71	-	.67	.71	.52	.61
UV6	.33	.19	.22	.37	.36	.35	.19	.21	.39	.29	.28	.38	.42	.40	.39	.28	.40	.50	.51	.59	.64	.58	-	.75	.59	.68
UV7	.39	.28	.29	.42	.40	.39	.25	.29	.44	.36	.34	.39	.46	.43	.41	.29	.45	.52	.53	.69	.73	.64	.71	-	.63	.70
UV8	.28	.19	.19	.31	.28	.30	.15	.17	.29	.18	.20	.26	.30	.27	.32	.22	.31	.37	.39	.54	.50	.43	.53	.56	-	.66
UV9	.40	.28	.28	.41	.37	.37	.24	.28	.44	.34	.36	.43	.46	.42	.47	.33	.45	.54	.45	.58	.55	.48	.62	.65	.64	-

Appendix C. (b) Correlation among Observed Items of the Three Science Motivational Beliefs in Australia

	SC1	SC2	SC3	SC4	SC5	SC6	SC7	SC8	IV1	IV2	IV3	IV4	IV5	IV6	IV7	IV8	IV9	UV1	UV2	UV3	UV4	UV5	UV6	UV7	UV8	UV9
SC1	-	.41	.50	.65	.65	.52	.40	.40	.57	.37	.44	.48	.59	.56	.45	.34	.59	.43	.36	.41	.39	.46	.40	.40	.34	.43
SC2	.51	-	.65	.41	.38	.22	.65	.57	.30	.39	.39	.24	.34	.30	.22	.17	.34	.20	.14	.22	.21	.26	.22	.24	.18	.23
SC3	.60	.66	-	.46	.48	.32	.61	.59	.43	.47	.49	.32	.46	.43	.28	.22	.49	.29	.22	.28	.29	.37	.28	.28	.21	.28
SC4	.64	.48	.56	-	.74	.52	.41	.44	.59	.40	.44	.51	.61	.59	.47	.36	.62	.45	.40	.45	.43	.50	.44	.43	.36	.47
SC5	.62	.46	.56	.71	-	.55	.38	.43	.57	.38	.42	.47	.59	.58	.44	.34	.61	.44	.41	.43	.44	.51	.43	.41	.33	.43
SC6	.52	.35	.42	.52	.54	-	.20	.25	.50	.29	.35	.42	.49	.52	.39	.27	.51	.38	.37	.35	.34	.39	.35	.33	.29	.36
SC7	.51	.64	.63	.51	.47	.34	-	.65	.35	.41	.41	.27	.37	.33	.24	.18	.37	.19	.14	.21	.19	.26	.20	.22	.16	.22
SC8	.51	.58	.61	.55	.52	.37	.66	-	.40	.43	.49	.30	.42	.39	.26	.19	.42	.25	.20	.24	.24	.32	.22	.23	.18	.24
IV1	.57	.39	.51	.59	.54	.46	.46	.50	-	.49	.60	.67	.83	.79	.61	.45	.77	.52	.44	.45	.44	.54	.49	.46	.38	.50
IV2	.45	.43	.52	.46	.45	.37	.48	.50	.63	-	.66	.41	.54	.52	.38	.23	.52	.36	.30	.34	.34	.39	.36	.32	.28	.38
IV3	.44	.40	.50	.48	.44	.36	.46	.52	.65	.70	-	.49	.62	.62	.44	.30	.62	.40	.33	.36	.35	.43	.38	.36	.30	.41
IV4	.46	.31	.40	.49	.43	.39	.37	.38	.67	.50	.54	-	.70	.67	.65	.47	.63	.50	.43	.45	.42	.44	.49	.46	.36	.51
IV5	.58	.41	.54	.60	.55	.46	.48	.52	.83	.66	.68	.69	-	.83	.62	.47	.81	.55	.46	.49	.48	.57	.52	.48	.41	.54
IV6	.54	.38	.52	.58	.55	.47	.45	.52	.79	.64	.67	.68	.84	-	.61	.45	.80	.54	.48	.50	.49	.56	.51	.48	.40	.53
IV7	.42	.27	.35	.44	.39	.37	.31	.34	.56	.44	.46	.61	.58	.57	-	.45	.56	.53	.45	.46	.43	.44	.54	.50	.43	.52
IV8	.25	.17	.23	.27	.28	.21	.20	.21	.40	.30	.30	.40	.41	.41	.39	-	.46	.32	.28	.31	.28	.31	.32	.33	.31	.38
IV9	.56	.40	.57	.59	.55	.46	.48	.52	.76	.62	.64	.60	.79	.80	.52	.39	-	.52	.46	.50	.50	.60	.50	.47	.39	.52
UV1	.38	.23	.33	.40	.40	.33	.27	.30	.50	.44	.42	.48	.52	.50	.52	.26	.49	-	.70	.65	.62	.60	.70	.64	.54	.65
UV2	.32	.17	.28	.36	.39	.34	.22	.23	.43	.38	.34	.40	.43	.43	.41	.21	.42	.70	-	.64	.61	.57	.63	.58	.49	.57
UV3	.33	.20	.30	.34	.35	.30	.22	.25	.41	.38	.33	.37	.45	.43	.40	.22	.43	.60	.61	-	.80	.69	.68	.69	.55	.67
UV4	.31	.20	.30	.33	.33	.29	.22	.24	.39	.35	.31	.34	.43	.42	.35	.23	.42	.58	.57	.86	-	.77	.67	.67	.54	.63
UV5	.39	.28	.41	.43	.43	.35	.32	.34	.51	.45	.42	.40	.54	.53	.38	.25	.55	.57	.54	.71	.78	-	.64	.62	.49	.58
UV6	.35	.21	.30	.36	.35	.31	.24	.26	.45	.41	.38	.45	.47	.46	.49	.26	.43	.66	.60	.60	.58	.57	-	.73	.59	.70
UV7	.34	.21	.31	.34	.34	.31	.25	.24	.40	.38	.34	.40	.44	.42	.46	.26	.41	.60	.58	.67	.67	.62	.70	-	.60	.69
UV8	.28	.15	.23	.27	.28	.26	.16	.18	.32	.29	.26	.30	.34	.33	.34	.23	.31	.46	.43	.49	.46	.42	.49	.53	-	.69
UV9	.39	.23	.33	.38	.36	.32	.25	.26	.47	.41	.40	.46	.50	.48	.49	.30	.45	.59	.54	.59	.56	.52	.62	.65	.65	-

Appendix C. (c) Correlation among Observed Items of the Three Science Motivational Beliefs in the United States

	SC1	SC2	SC3	SC4	SC5	SC6	SC7	SC8	IV1	IV2	IV3	IV4	IV5	IV6	IV7	IV8	IV9	UV1	UV2	UV3	UV4	UV5	UV6	UV7	UV8	UV9
SC1	-	.34	.35	.66	.62	.50	.33	.31	.55	.27	.33	.43	.54	.49	.43	.41	.51	.38	.31	.35	.33	.37	.36	.36	.36	.44
SC2	.47	-	.62	.35	.31	.15	.67	.58	.25	.39	.41	.17	.29	.23	.21	.18	.26	.10	.05	.13	.10	.14	.15	.15	.13	.17
SC3	.45	.65	-	.36	.34	.22	.59	.54	.31	.40	.43	.21	.35	.30	.22	.20	.37	.18	.13	.18	.15	.22	.20	.19	.16	.20
SC4	.63	.48	.49	-	.73	.53	.36	.36	.57	.29	.35	.46	.57	.54	.43	.43	.54	.37	.31	.34	.33	.38	.37	.37	.32	.40
SC5	.57	.39	.45	.69	-	.52	.30	.33	.52	.27	.31	.41	.54	.52	.41	.41	.54	.39	.35	.34	.34	.41	.39	.38	.33	.39
SC6	.49	.30	.34	.53	.53	-	.13	.18	.46	.23	.28	.39	.45	.46	.38	.33	.47	.36	.34	.31	.30	.33	.35	.31	.31	.35
SC7	.47	.69	.63	.46	.38	.26	-	.66	.29	.39	.42	.19	.31	.25	.21	.19	.30	.13	.05	.13	.10	.13	.15	.16	.13	.18
SC8	.42	.58	.59	.46	.42	.28	.67	-	.29	.39	.44	.19	.31	.27	.18	.16	.30	.15	.08	.13	.10	.17	.16	.15	.12	.16
IV1	.52	.37	.45	.55	.51	.44	.40	.40	-	.38	.49	.64	.80	.76	.58	.54	.74	.49	.40	.41	.39	.46	.44	.40	.34	.47
IV2	.37	.42	.47	.39	.35	.28	.42	.42	.51	-	.62	.27	.40	.38	.26	.21	.39	.27	.21	.22	.19	.25	.24	.22	.15	.24
IV3	.36	.38	.45	.39	.37	.32	.40	.45	.55	.63	-	.39	.52	.49	.34	.29	.51	.31	.26	.27	.25	.32	.28	.26	.19	.29
IV4	.37	.25	.30	.44	.37	.33	.27	.25	.63	.40	.43	-	.68	.66	.63	.50	.60	.44	.37	.39	.36	.37	.43	.39	.33	.45
IV5	.52	.39	.47	.58	.53	.44	.41	.42	.80	.54	.58	.67	-	.79	.60	.55	.77	.47	.39	.40	.40	.47	.45	.41	.35	.46
IV6	.45	.32	.43	.53	.50	.44	.35	.38	.74	.52	.55	.64	.81	-	.60	.52	.76	.49	.45	.42	.42	.49	.46	.42	.34	.45
IV7	.37	.24	.28	.40	.35	.34	.24	.23	.53	.37	.37	.58	.57	.55	-	.52	.55	.48	.40	.43	.38	.38	.48	.46	.40	.49
IV8	.33	.22	.25	.35	.32	.25	.23	.21	.46	.29	.30	.47	.50	.48	.45	-	.54	.34	.31	.32	.30	.32	.33	.36	.34	.40
IV9	.47	.37	.49	.56	.52	.43	.40	.41	.74	.52	.56	.58	.78	.76	.51	.47	-	.46	.40	.40	.40	.51	.43	.40	.33	.43
UV1	.33	.16	.24	.35	.36	.31	.18	.22	.48	.36	.37	.42	.48	.48	.49	.29	.44	-	.69	.59	.57	.54	.66	.60	.46	.55
UV2	.26	.11	.19	.31	.33	.31	.12	.18	.40	.28	.28	.35	.41	.44	.40	.27	.39	.66	-	.58	.59	.52	.60	.55	.44	.49
UV3	.29	.16	.21	.30	.29	.26	.16	.17	.35	.25	.26	.32	.37	.37	.38	.27	.34	.54	.54	-	.71	.58	.66	.68	.53	.59
UV4	.27	.14	.21	.29	.29	.26	.15	.17	.36	.27	.27	.32	.38	.40	.33	.26	.37	.52	.50	.73	-	.73	.65	.67	.47	.54
UV5	.32	.20	.30	.39	.37	.31	.22	.24	.45	.34	.36	.36	.48	.48	.35	.27	.50	.50	.44	.57	.74	-	.61	.60	.40	.48
UV6	.29	.16	.23	.33	.32	.31	.18	.18	.40	.30	.30	.39	.41	.43	.45	.29	.39	.62	.57	.62	.60	.56	-	.71	.55	.63
UV7	.31	.17	.22	.33	.31	.28	.18	.18	.38	.27	.28	.33	.39	.38	.41	.29	.36	.56	.51	.66	.66	.59	.67	-	.58	.64
UV8	.28	.11	.17	.27	.24	.26	.13	.12	.29	.21	.20	.29	.31	.28	.35	.25	.25	.43	.38	.48	.43	.34	.48	.49	-	.68
UV9	.35	.17	.23	.33	.30	.29	.21	.20	.41	.29	.29	.40	.43	.40	.43	.34	.36	.51	.44	.56	.50	.43	.58	.60	.62	-

Notes. All coefficients are significant at $p < .001$. Lower triangle: Female; Upper triangle: Male.

Appendix D. Measurement Equivalence for Science Motivational Beliefs by Gender in Australia and the United States

The MI of the Australian adolescents' science motivational beliefs reached invariant uniqueness. The fit indices for M1 were poor, χ^2 (296, N = 8849) = 9654.11, RMSEA = .060, CFI = .873, TLI = .860, and SRMR = .060. M2 was improved after taking into account the effect of negative wording of the items, χ^2 (296, N = 8849) = 5788.99, RMSEA = .046, CFI = .925, TLI = .916, and SRMR = .046. It is concluded that the three-factor model was supported. Moreover, the results also indicate that the negatively worded items substantially impacted the model's fit. The results of M3 indicate that the factorial construct was equal across genders, χ^2 (578, N = 8849) = 10412.06, RMSEA = .062, CFI = .925, TLI = .915, and SRMR = .047. Subsequently, an examination of metric invariance (M4) was performed by constraining the factor loading of males and females to be equal. The testing difference of the fit indexes found that the factor loadings were equal across groups (ΔRMSEA = 0, ΔCFI = .002). Scalar invariance (M5) was established by constraining the intercepts for each item to be equal across groups. Based on the criteria, the results indicated that scalar invariance was present in the data (ΔRMSEA = 0, ΔCFI = .003). Invariant uniqueness (M6) was established by constraining the unique variances for each item to be equal across groups. The findings suggested that all items attained invariant uniqueness (ΔRMSEA < .001, ΔCFI =.001).

The MI of the US adolescents' science motivational beliefs reached invariant uniqueness. The fit indices for M1 were poor, χ^2 (296, N = 8252) = 11908.04, RMSEA = .069, CFI = .806, TLI = .787, and SRMR = .070. M2 was improved after taking into account the effect of negative wording of the items. χ^2 (289, N = 8252) = 5526.68, RMSEA = .047, CFI = .912, TLI = .901, and SRMR = .052. It is concluded that the three-factor model was supported. Moreover, the results also indicate that the negatively worded items

substantially impacted the model's fit. The results of M3 indicate that the factorial construct was equal across genders (χ^2 (578, N = 8252) = 8651.07, RMSEA = .058, CFI = .923, TLI = .913, and SRMR = .053). Subsequently, an examination of metric invariance (M4) was performed by constraining the factor loading of males and females to be equal. The testing difference of the fit indexes found that the factor loadings were equal across groups (ΔRMSEA = 0, ΔCFI = .003). Scalar invariance (M5) was established by constraining the intercepts for each item to be equal across groups. Based on the criteria, the results indicated that scalar invariance was present in the data (ΔRMSEA = 0, ΔCFI = .004). Invariant uniqueness (M6) was established by constraining the unique variances for each item to be equal across groups. The findings suggested that all items attained invariant uniqueness (ΔRMSEA <.001, ΔCFI= 0).
